# Tadalafil Versus Tamsulosin or Silodosin as Medical Expulsive Therapy for Distal Ureteral Stones: A Systematic Review and Meta-Analysis of Randomized Controlled Trials

**DOI:** 10.5152/tud.2025.24145

**Published:** 2025-12-05

**Authors:** Murilo Ribeiro Sanches, Lucas Guimarães Campos Roriz de Amorim, Marcus Vinícius Barbosa Moreira, Lucas Bresciani Padilha, Lígia Sant’Ana Dumont, Edoardo Pozzi, Francesco Costantini Mesquita, Lucas Ribeiro Campos, Marcelo Esteves Chaves Campos, Ranjith Ramasamy

**Affiliations:** 1 Department of Medicine, Federal University of Goiás, Goiânia, Brazil; 2 Department of Urology, Federal University of Minas Gerais, Belo Horizonte, Brazil; 3 Department of Medicine, Potiguar University, Natal, Brazil; 4 Department of Medicine, Federal University of Rio de Janeiro, Rio de Janeiro, Brazil; 5 Department of Medicine, Evangelical University of Goiás, Anápolis, Brazil; 6 Department of Urology, University of Miami Miller School of Medicine, Miami, United States of America; 7 Department of Urology, University Vita-Salute San Raffaele, Milan, Italy; 8 Department of Urology, São José do Rio Preto School of Medicine (FAMERP/FUNFARME), São José do Rio Preto, São Paulo, Brazil; 9 Department of Urology, Jumeirah American Clinic, Dubai, UAE

**Keywords:** Adrenergic alpha-1 receptor antagonists, meta-analysis, phosphodiesterase 5 inhibitors, tadalafil, ureteral calculi

## Abstract

**Objective:**

Medical expulsive therapy (MET) facilitates the passage of distal ureteric stones. Alpha-blockers are the standard MET strategy. However, there has been growing interest in using tadalafil, a Phosphodiesterase type 5 (PDE5) inhibitor, to facilitate ureteral stone passage. Therefore, a systematic review and meta-analysis comparing tadalafil versus tamsulosin or silodosin was conducted as MET options for patients with distal ureteral stones.

**Methods:**

PubMed, Embase, and Cochrane Library were systematically searched in September 2023 for randomized controlled trials (RCTs) comparing tadalafil with alpha-blockers (tamsulosin or silodosin) for distal ureteric stones. Outcomes included stone expulsion rate (SER), stone expulsion time (SET), colic episodes, analgesic use, and side effects. Review Manager 5.4.1 was used for statistical analysis, applying a random-effects model.

**Results:**

Eleven RCTs were included with 1345 patients, 579 (43%) randomized to tadalafil. No significant differences were found between groups for SER (risk ratio [RR] 1.07; 95% CI 0.98-1.18; *P* = .14), SET (RR −0.68; 95% CI −1.75 to 0.38; *P* = .21), colic episodes, or analgesic use. Headaches were significantly less frequent with alpha-blockers (RR 1.50; 95% CI 1.09-2.04; *P* = .01), while abnormal ejaculation was significantly less frequent in the tadalafil group (RR 0.38; 95% CI 0.19-0.74; *P* = .005).

**Conclusion:**

Given the comparable efficacy in stone expulsion and the distinct side-effect profiles, the choice between tadalafil and alpha-blockers for MET can be individualized. Tadalafil emerges as a strong MET alternative, particularly when alpha-blockers are contraindicated or poorly tolerated.

## Introduction

Urinary stone disease constitutes a highly impactful condition, affecting 2%-3% of the general population, and carries a substantial risk of recurrence.^1^ While studies indicate that 71%-98% of stones smaller than 5 mm will pass spontaneously, only 25%-51% of stones sized 5-10 mm exhibit spontaneous passage. Stones that fail to pass may cause prolonged colic episodes and heightened risks of complications.^2^^-^^4^

Management of acute stone-related colic focuses on analgesia, treatment of suspected or confirmed infection, and prompt relief of obstruction.^1^ Various urological strategies can be employed for ureteral stones depending on the clinical presentation and stone dimensions, including observation, medical expulsive therapy (MET), drainage, extracorporeal shock wave lithotripsy, and ureteroscopy.^5^

Medical expulsion therapy was designed to facilitate ureteral stone passage and shorten stone expulsion time (SET) post-lithotripsy.^6^ Given that a substantial proportion of stones exhibit spontaneous passage and surgical operative interventions carry procedure‑related morbidity, current management of uncomplicated ureteral stones ≤10 mm has increasingly shifted toward MET.^7^^-^^9^

According to the most recent European Association of Urology guidelines, alpha-blockers, such as tamsulosin and silodosin, are the preferred MET option for patients with distal ureteral stones >5 mm who are suitable for conservative management.^6^ These agents relax distal ureteral smooth muscle via alpha-1 adrenergic blockade (particularly the 1A/1D subtypes), reducing spasm and intraluminal pressure to facilitate stone passage.^10^^-^^13^ However, additional drug classes, including calcium channel blockers, corticosteroids, nonsteroidal anti-inflammatory drugs, terpene compound products, plant extracts, and phosphodiesterase type 5 inhibitors (PDEI-5), have also been investigated as potential MET agents.^10^^-^^12^^,^^14^

Among these, tadalafil, a PDEI-5, has emerged as a compelling option, augmenting Nitric Oxide/Cyclic Guanosine Monophosphate signaling to induce ureteral smooth muscle relaxation.^15^ Prior meta-analyses have explored the effect of tadalafil as MET.^16^^-^^18^ However, no definitive recommendations can be established due to the limited number of studies included. In a recent meta-analysis, Belkovsky et al^18^ reported a significantly higher stone expulsion rate (SER) with tadalafil compared to tamsulosin, with no differences in SET or adverse events. Notably, only 7 of the included studies were peer‑reviewed articles, while the remaining 4 were conference abstracts, which constrains quality appraisal and the certainty of the evidence.

Moreover, despite supportive evidence for silodosin as an effective MET, none of the previous head-to-head meta-analyses included silodosin in their comparisons.^16^^-^^21^ Therefore, to address this gap and provide updated evidence on this topic, a systematic review and meta-analysis comparing the efficacy and safety of tadalafil versus tamsulosin or silodosin as MET for patients with distal ureteral stones was conducted.

## Materials and Methods

### Protocol and Registration

This systematic review followed the guidelines established by the Preferred Reporting Items for Systematic Reviews and Meta-Analyses (PRISMA).^22^ The protocol was registered prospectively with the International Prospective Register of Systematic Reviews under registration number CRD42023466555.

### Eligibility Criteria

Studies that met the following eligibility criteria were included: randomized controlled trials (RCTs); comparing tadalafil versus alpha-blockers; for distal ureteral stones; in patients >18 years; reporting any of the outcomes of interest. No language or sample-size restrictions were applied.

Exclusion criteria encompassed any study that did not align with the PICOT framework as follows: (P) population: patients >18 years with distal ureteral stones (ureterolithiasis); (I) intervention: tadalafil as METs; (C) control: alpha-blockers (tamsulosin and/or silodosin) as METs; (O) outcomes: SER, SET, analgesic use, colic episodes, headache, dizziness, back pain, orthostatic hypotension, and abnormal ejaculation; (T) type of studies: RCTs. Studies with non-randomized or single-arm designs were specifically excluded; animal studies, case reports, or case series; non-full-text publications (conference abstracts); and trials using other combined therapies that could confound treatment effects.

### Search Strategy and Data Extraction

We systematically searched PubMed, Embase, and Cochrane Central Register of Controlled Trials in September 2023 for studies that met the inclusion criteria. The following search terms were used: (tadalafil OR “phosphodiesterase type 5 inhibitors” OR PDE5i OR “PDE5 inhibitors” OR “Phosphodiesterase 5 inhibitors”) AND (“alpha-blocker” OR “α-blocker” OR tamsulosin OR silodosin) AND (stone OR “expulsive therapy” OR nephrolithiasis OR urolithiasis OR calculi).

All identified articles were systematically assessed using the predefined criteria mentioned above. Two authors (M.R.S. and L.B.P.) independently conducted the screening and selection processes. Disagreements were addressed and resolved through consensus among the authors. Additionally, backward snowballing was employed to identify further relevant studies.

Two authors (M.R.S. and M.V.B.M.) independently extracted baseline characteristics and outcome data from the selected studies, using a standardized data extraction form. Any discrepancies were settled by consensus between the authors.

### Endpoints and Definitions

The primary endpoints of interest were SER and SET. The secondary outcomes were analgesic use, colic episodes, headache, dizziness, back pain, orthostatic hypotension, and abnormal ejaculation.

Stone expulsion rate was defined as the proportion of participants who achieved complete passage of the distal ureteral stone. In each study, it was assessed at the end of the prespecified MET course, which varied between 2 and 4 weeks. The imaging method used to confirm the stone passage also varied between studies (radiography, ultrasonography, or computed tomography scan), with each study using the same modality applied at baseline. On the other hand, SET was defined as the interval from the initiation of MET use to the first documented evidence of passage, based on patient-reported stone capture or imaging confirmation.

### Quality Assessment

The risk of bias for each RCT was assessed using the Cochrane Collaboration’s tool for evaluating bias in randomized trials (RoB 2), in accordance with the Cochrane Handbook for Systematic Reviews of Interventions.^23^^,^^24^ Each trial was assigned a bias risk rating, indicating whether it posed high, some concerns, low, or unclear risk of bias across 5 areas: randomization process, deviations from intended interventions, missing outcome data, outcome measurement, and selective reporting. Two authors (M.R.S. and L.A.) conducted this assessment independently, and any disagreements were resolved by reaching a consensus.

To assess small study effects (publication bias), a contour-enhanced funnel plot analysis was utilized for the SER outcome, examining for symmetry in the distribution of trial weights.^25^ Egger’s regression test was subsequently performed, and a leave-one-out sensitivity analysis was conducted to identify the impact of individual studies on the overall results.^26^

### Statistical Analysis

All statistical analyses were conducted following Cochrane recommendations.^24^ The Mantel–Haenszel method was used to calculate pooled risk ratios (RRs) with 95% CIs for binary outcomes, while the inverse-variance method was employed to pool mean differences (MDs) for continuous outcomes, utilizing means ± SDs. For continuous outcomes where RCTs reported only medians (interquartile ranges), the corresponding means ± SDs were estimated using the method proposed by Wan and Luo.^27^^,28^ Prespecified subgroup analyses were conducted based on the tadalafil dosage (5 mg versus 10 mg) for the primary endpoints (SER and SET), with significance for subgroup differences considered when *P*-values were less than .05.

Cochran’s Q test and the *I*² statistic were applied to evaluate between-study heterogeneity; significance was determined at *P*-values less than .10 and *I*² > 25%. The DerSimonian and Laird random-effects model was used to account for demographic variability across studies. Primary statistical analyses were performed using Review Manager 5.4.1 (Cochrane Centre, The Cochrane Collaboration, Denmark), and R software 4.2.2 (R Foundation for Statistical Computing, Austria) was employed specifically for Egger’s regression test.

## Results

### Study Selection and Characteristics

**Figure 1.**
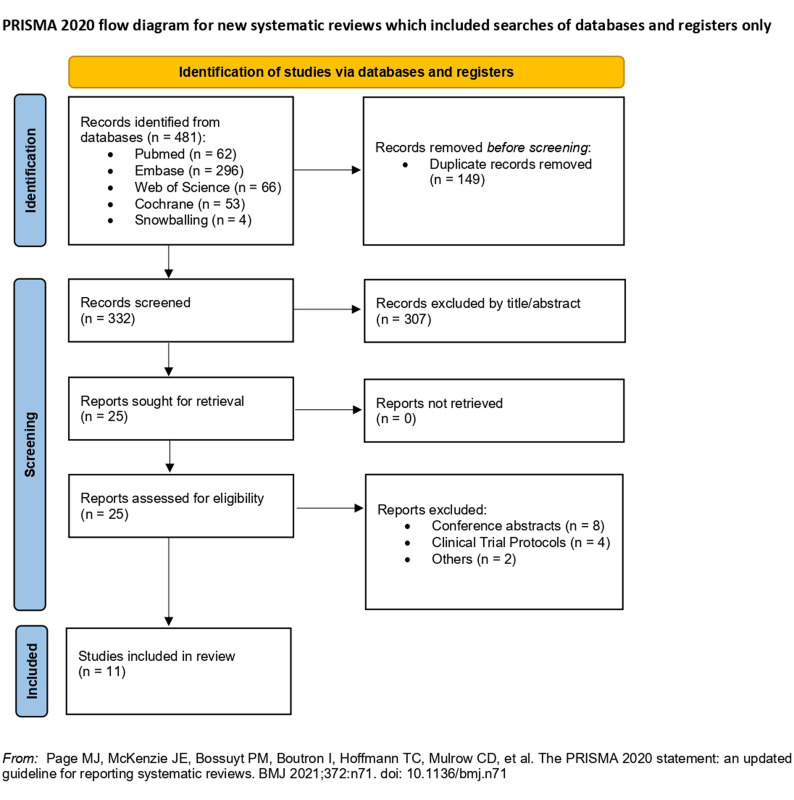
Preferred Reporting Items for Systematic Reviews and Meta-Analyses flow diagram of study screening and selection.

As detailed in Figure 1, the initial search yielded 481 results. After the removal of duplicate records and the assessment of the studies based on title and abstract, 25 studies remained for full-text review according to prespecified criteria. Of these, 11 RCTs were included, comprising 1345 patients, of whom 579 (43%) were randomized to tadalafil.^2^^-^^4^^,^^29^^-^^36^

Individual study characteristics are detailed in Table 1. The mean age ranged from 32.05 to 45.38 years in the tadalafil group and ranged from 31.37 to 43.18 years in the alpha-blockers group. Treatment duration ranged from 2 to 4 weeks. Mean stone size in tadalafil and alpha-blockers groups was 6.64 and 6.67 mm, respectively. In 4 studies, patients used 5 mg of tadalafil daily, while in the other 7 studies, patients used 10 mg daily. All studies utilized tamsulosin as the alpha-blocker of choice for comparison with tadalafil; however, silodosin was also assessed in 3 studies.

### Pooled Analysis of All Studies

**Figure 2.**
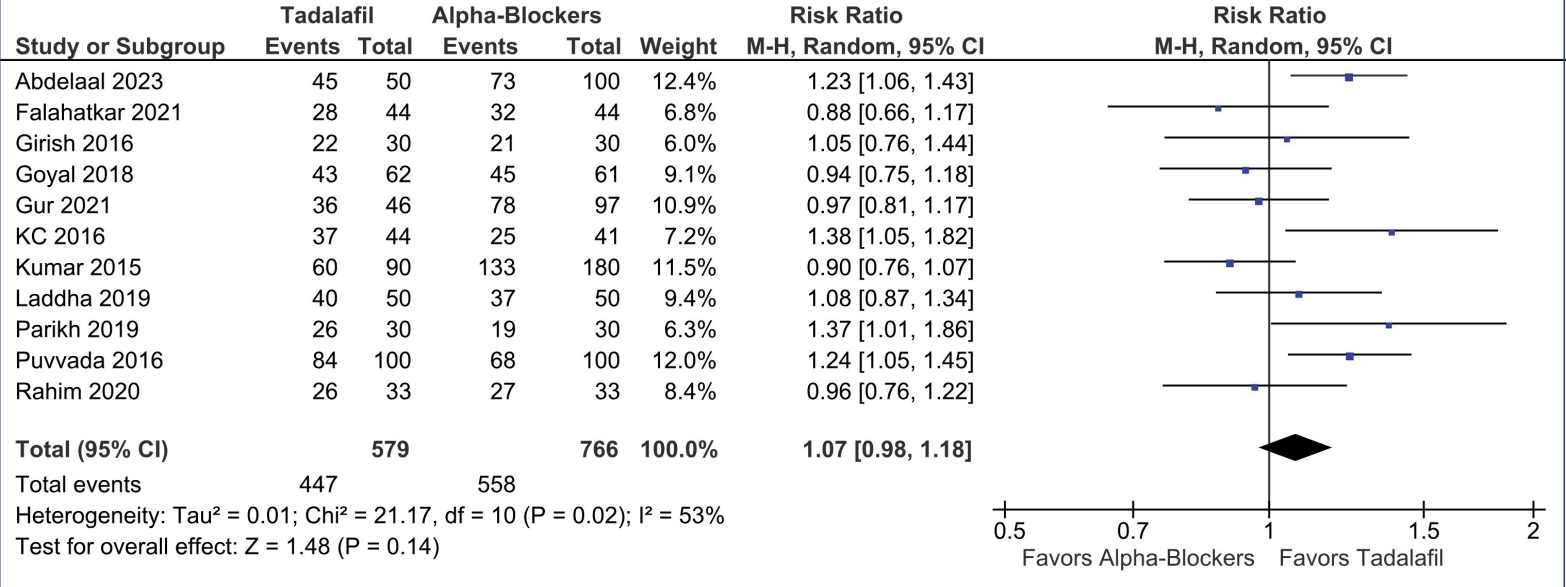
There was no significant difference between groups in terms of stone expulsion rate (*P* = .14).

**Figure 3.**
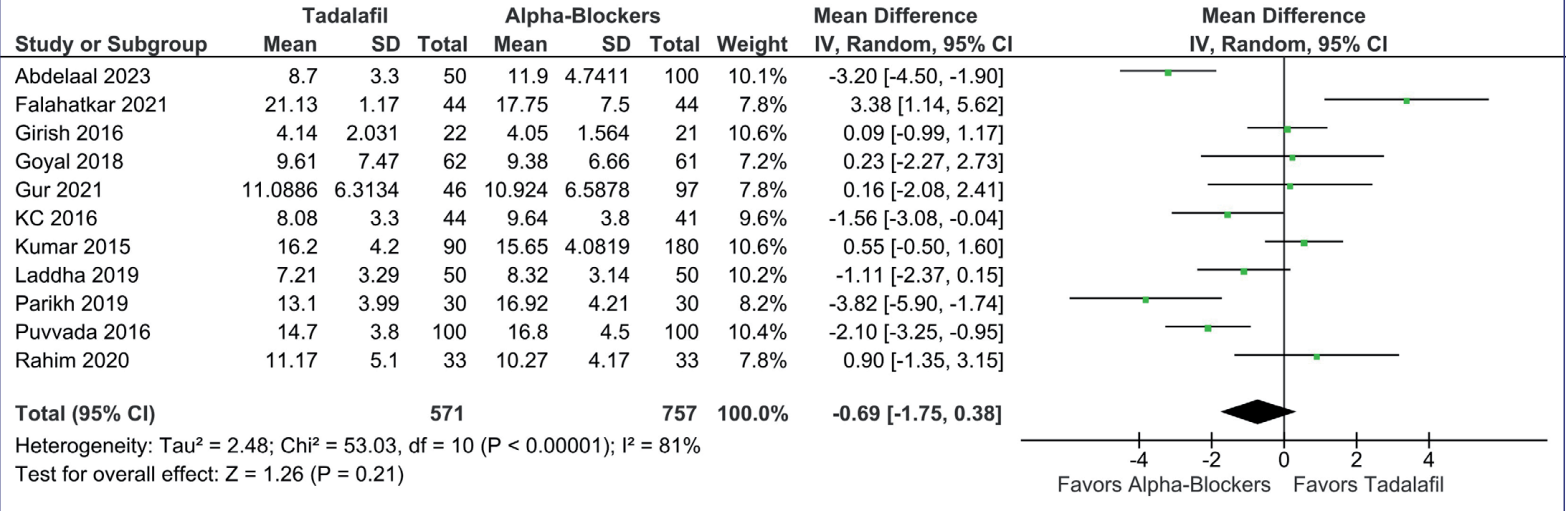
There was no significant difference between groups in terms of stone expulsion time (*P* = .21).

There was no significant difference between groups in terms of SER (RR 1.07; 95% CI 0.98-1.18; *P* = .14; *I*² = 53%; Figure 2), and SET (RR −0.69; 95% CI −1.75 to 0.38; *P* = .21; *I*² = 81%; Figure 3).

**Figure 4.**
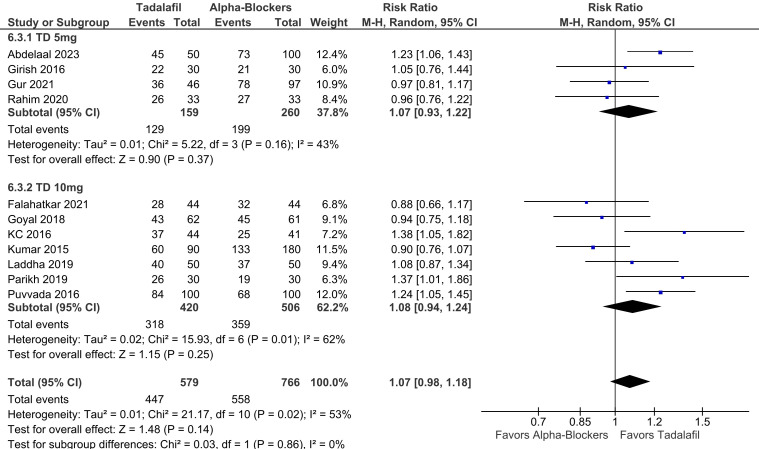
There was no significant difference between groups in terms of colic episodes (*P* = .41).

**Figure 5.**
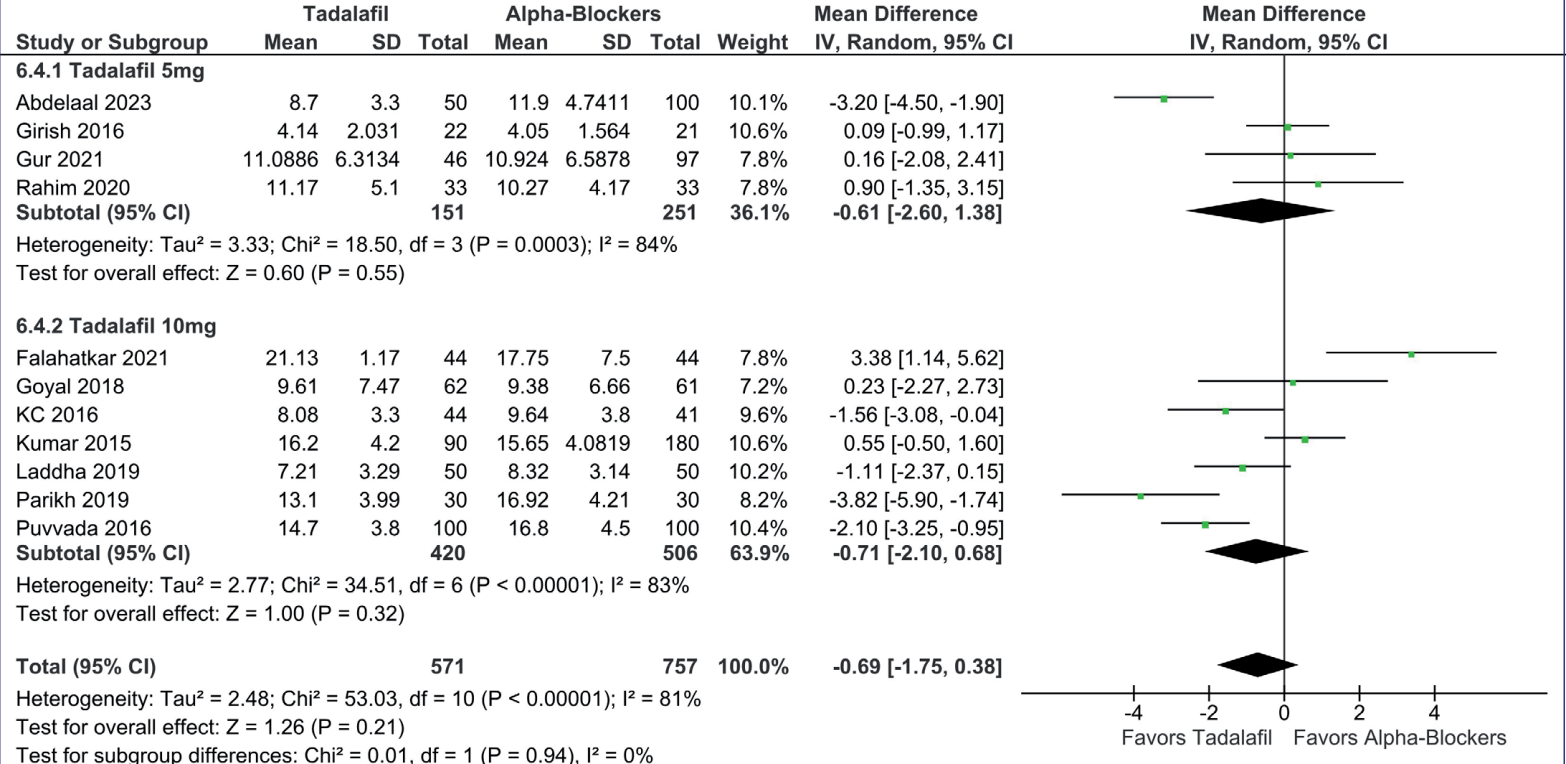
There was no significant difference between groups in terms of analgesic use (*P* = .21).

There was also no difference between tadalafil and alpha-blockers in terms of colic episodes (MD −0.25 episodes; 95% CI −0.85 to 0.34; *P* = .41; *I*² = 95%; Figure 4) and analgesic use (MD −43.08 mg; 95% CI 111.13-24.97; *P* = .21; *I*² = 95%; Figure 5).

In terms of safety endpoints, headache was significantly higher in the tadalafil group (RR 1.50; 95% CI 1.09-2.04; *P* = .01; *I*² = 0%; Supplementary Figure 1), and abnormal ejaculation was significantly lower in the tadalafil group (RR 0.36; 95% CI 0.22-0.59; *P* < .001; *I*² = 0%; Supplementary Figure 2). There were no differences between groups in dizziness (RR 1.32; 95% CI 0.91-1.93; *P* = .14; *I*² = 0%; Supplementary Figure 3), back pain (RR 1.22; 95% CI 0.79-1.87; *P* = .37; *I*² = 25%; Supplementary Figure 4), and orthostatic hypotension (RR 0.89; 95% CI 0.56-1.41; *P* = .61; *I*² = 18%; Supplementary Figure 5).

### Subgroup Analysis

**Figure 6.**
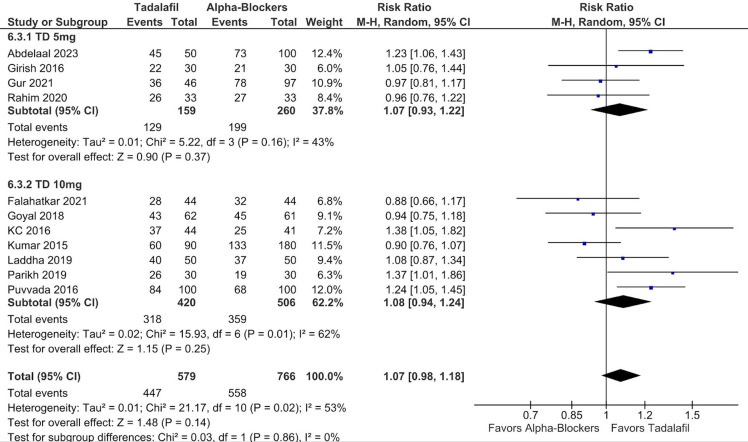
There was no significant interaction between subgroups stratified according to tadalafil dosage (5 mg and 10 mg) in stone expulsion rate (p-interaction = 0.86).

**Figure 7.**
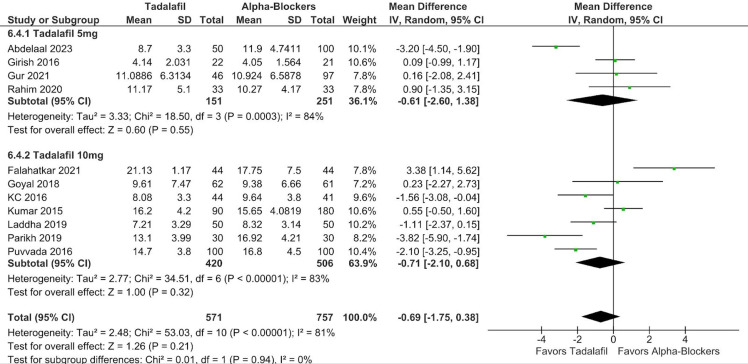
There was no significant interaction between subgroups stratified according to tadalafil dosage (5 mg and 10 mg) in stone expulsion time (p-interaction = 0.94).

In subgroup analysis stratified according to tadalafil dosage, there were no significant differences between groups in SER (RR 1.07; 95% CI 0.93-1.22; *P* = .37; *I*² = 43%; Figure 6) and SET (RR −0.61; 95% CI −2.60 to 1.38; *P* = .55; *I*² = 84%; Figure 7) in patients using 5 mg. There were also no differences between groups in SER (RR 1.08; 95% CI 0.94-1.24; *P* = .25; *I*² = 62%; Figure 6), and SET (RR −0.71; 95% CI −2.10 to 0.68; *P* = .32; *I*² = 83%; Figure 7) in patients using 10 mg. No significant interaction between subgroups was observed for SER or SET, with a *P*-value for subgroup differences of .86 and .94, respectively.

### Quality Assessment

Individual RCT appraisals, conducted in accordance with the Cochrane Collaboration’s RoB 2 tool, are presented in Supplementary Figure 6. One study was categorized as high risk due to the randomization process based on odd and even numbers.^2^ Furthermore, 3 studies raised some concerns because of high follow-up attrition rates.^29^^,^^33^^,^^34^ Additionally, 1 study was assessed as raising concerns owing to notable differences in stone size and density between groups, which raised potential issues about differing prognoses across groups.^4^

The funnel plot analysis for the SER outcome indicated no evidence of small study effects (publication bias), as shown in Supplementary Figure 7. The studies displayed a symmetrical distribution by weight, trending toward the pooled effect size as weight increased. Egger’s regression test supported this result, with no indication of publication bias (*P* = .83). Sensitivity analyses employing the leave-one-out method yielded results consistent with the overall pooled analysis across all studies.

## Discussion

In this systematic review and meta-analysis involving 11 studies and 1345 patients with distal ureteric stones, tadalafil was compared with tamsulosin or silodosin. The primary findings from the pooled analyses were as follows: no significant differences were observed between groups in SER, SET, colic episodes, and analgesic use; the incidence of headaches was higher in the tadalafil group; abnormal ejaculation occurred less frequently in the tadalafil group; and no significant differences were found in subgroup analyses.

The management of uncomplicated ureteral stones ≤10 mm has evolved toward MET to facilitate stone expulsion, since spontaneous passage is less likely to occur as stone size increases.^7^^-^^9^ Prior network meta-analyses comparing the efficacy of multiple drug classes as METs suggest that tadalafil plus an alpha-blocker may be the most effective regimen, though the limited number of studies restricts the generalizability of the findings.^37^^-^^39^ Among single-drug regimens, alpha-blockers appear to be the most effective monotherapy.

While alpha-blockers have become the guideline-recommended standard of care, tadalafil, a PDEI-5, has emerged as a compelling alternative.^15^ Although previous network meta-analyses did not endorse tadalafil as the preferred individual therapy, it is noteworthy that only a restricted number of trials assessing tadalafil as monotherapy were included, leaving its comparative efficacy versus alpha-blockers unresolved.^37^^-^^39^

Head-to-head meta-analyses on the topic have also yielded conflicting results. Cardona et al^16^ conducted the first pairwise meta-analysis on the efficacy and safety of PDEI-5 for ureteric stones. The study primarily focused on tadalafil’s efficacy compared to placebo, with only a subset analysis of 2 studies directly comparing tadalafil against tamsulosin. While no differences were found between tadalafil and tamsulosin groups, this meta-analysis was the pioneer in establishing the benefits of tadalafil compared to placebo in ureteric stone management.^16^ Subsequently, Bai et al^17^ reported significant differences favoring the tadalafil group in SER, SET, and analgesic use, with no differences between groups in terms of pain episodes and complications. However, their results were based on only 4 studies. Lastly, Belkovsky et al^18^ found a significant difference favoring tadalafil in SER, with comparable results in terms of SET, side effects, colic episodes, and analgesic use. However, the inclusion of conference abstracts hindered a comprehensive appraisal of evidence quality. Overall, direct comparisons remain inconsistent and constrained by few trials and variable study quality.

More recently, Sharma et al^38^ conducted an updated network meta-analysis of 50 RCTs (12 382 patients) and found silodosin to be the most effective monotherapy for MET. Although tamsulosin and silodosin share mechanistic similarities and current guidelines do not endorse a specific alpha-blocker, prior pairwise meta-analyses have largely centered on tamsulosin.^16^^-^^18^ Therefore, the aim was to assess tadalafil against alpha-blockers as a class, incorporating both tamsulosin and silodosin, for the management of distal ureteral stones.

In contrast to previous comparative meta-analyses that reported higher SER for the tadalafil group over tamsulosin, this study revealed no significant differences between therapies in either SER or SET.^16^^-^^18^ This discrepancy may arise from the incorporation of silodosin, as multiple RCTs suggest that silodosin achieves higher success rates when compared with tamsulosin or tadalafil.^19^^-^^21^^,^^33^^,^^35^ Additionally, this dataset included more patients, thereby enhancing statistical power. Moreover, inclusion was limited to full-text RCTs, improving methodological rigor. Altogether, these methodological enhancements strengthen the robustness and reliability of this updated meta-analysis.

In the subgroup analysis stratified by varying dosages of tadalafil, a prior meta-analysis indicated that tadalafil 5 mg increased SER compared to tamsulosin, while 10 mg did not show a similar effect.^18^ Conversely, in the current analysis, no significant differences were observed between groups in SER or SET across different dosages, revealing no substantial interaction between subgroups based on tadalafil dosage regarding SER (p-interaction = 0.86) and SET (p-interaction = 0.94).

Regarding colic episodes and analgesic use, both agents are thought to attenuate the frequency and amplitude of phasic peristaltic contractions accompanying ureteric obstruction, consequently reducing analgesic requirements. Although studies suggest that alpha-blockade alleviates ureteric colic by blocking the C-fibers responsible for mediating pain, no differences were found between groups in these outcomes.^40^

Our analysis confirmed distinct, clinically relevant adverse effect profiles for each drug. Tadalafil was associated with a significantly higher incidence of headache, making alpha‑blockers a more attractive option for patients with a migraine history or poor tolerance to this adverse effect. Conversely, the lower frequency of abnormal ejaculation with tadalafil—a recognized side effect of alpha-blockers—renders it a valuable alternative for sexually active men concerned about this specific adverse event.^33^ Additionally, alpha-blockers are contraindicated in men with cataracts. In a recent population-based retrospective cohort of older men undergoing cataract surgery, tamsulosin exposure was significantly associated with serious ophthalmic complications, including intraoperative floppy iris syndrome (characterized by a flaccid, billowing iris; iris prolapse through surgical incisions; and progressive intraoperative miosis).^41^^,^^42^

We observed elevated *I*² indexes in SER and SET outcomes. Differences in follow-up duration likely contributed to that variability, as longer follow-up tends to increase both measures. Additionally, the imaging modality used to confirm stone expulsion may also influence SER. For SET, some studies instructed patients to increase fluid intake and use a urine strainer, introducing patient-dependent factors beyond investigator control, and potentially increasing the risk of reporting bias. Notably, the *I*² index quantifies the impact of heterogeneity on the effect estimate, rather than heterogeneity itself, and an elevated *I*² index by itself does not preclude conducting a pooled analysis.^43^ Of note, leave-one-out sensitivity analyses were conducted and demonstrated that the effect remained consistent regardless of the influence of individual studies.

This study has limitations. First, most trials were conducted in the Middle East, which may potentially introduce regional and ethnic bias. Additional multicenter, well-designed RCTs in diverse populations are needed to improve generalizability. Second, variation in treatment duration and tadalafil dosage across the included studies may have influenced outcomes. Thus, a subgroup analysis was conducted to mitigate its potential impact. Despite these limitations, the study has notable strengths. Methodological rigor was ensured through strict adherence to the PRISMA guidelines and a robust quality assessment using the Cochrane RoB 2 tool. Moreover, the inclusion of 11 RCTs comprising 1345 participants provides substantial statistical power to detect between-group differences.

In this meta-analysis of RCTs involving patients with ureteral stones, tadalafil and alpha-blockers showed no significant differences in SER, SET, incidence of renal colic, or analgesic use. Among the side effects, headaches were less frequent with alpha-blockers, whereas abnormal ejaculation occurred less often in the tadalafil group. While alpha-blockers remain the first-line MET, tadalafil could serve as an effective alternative, especially when alpha-blockers are contraindicated or not tolerated, supporting an individualized, patient-centered approach. Further high-quality trials are needed to clarify comparative effectiveness and safety and to guide patient selection.

## Supplementary Materials

Supplementary Material

## Figures and Tables

**Table 1.** Individual Characteristics of the Included Studies

Study	Country	Patients, n	Intervention Group	Control Group	Treatment Duration, Weeks	Male, n	Age^†^, y	Stone Size^†^, mm	Included Population
Abdelaal & El-Dydamony,^29^ 2023	Egypt	TD: 50 AB: 100	TD 5 mg	Tamsulosin 0.4 mg Silodosin 8 mg	4 weeks	TD: 34 AB: 67	TD: 41.9 AB: 40.0	TD: 6.9 ± 1.5 AB: 6.8 ± 1.453	Patients aged 20-60 years, with a single distal ureteric stone sized 5-10 mm, diagnosed by USG, X-ray, or CT KUB
Falahatkar et al,^30^ 2021	Iran	TD: 44 AB: 44	TD 10 mg	Tamsulosin 0.4 mg	4 weeks	TD: 23 AB: 24	TD: 37.4 ± 12 AB: 37.0 ± 11.35	TD: 6.9 ± 1.65 AB: 6.9 ± 1.46	Patients aged 18-64 years with renal colic and a single distal ureteric stone <10 mm
Girish et al,^31^ 2016	India	TD: 30 AB: 30	TD 5 mg	Tamsulosin 0.4 mg	4 weeks	TD: 18 AB: 20	NA	TD: 6.3 AB: 6.0	Patients with distal ureteric stones sized 5-10 mm
Goyal et al,^32^ 2018	India	TD: 62 AB: 61	TD 10 mg	Tamsulosin 0.4 mg	4 weeks	TD: 41 AB: 43	TD: 42.6 ± 14.93 AB: 42.1 ± 13.18	TD: 7.6 ± 0.91 AB: 7.5 ± 1.11	Patients aged >18 years, with distal ureteric stones sized 6-10 mm
Gur et al,^33^ 2021	Türkiye	TD: 46 AB: 97	TD 5 mg	Tamsulosin 0.4 mg Silodosin 8 mg	4 weeks	TD: 46 AB: 97	TD: 39.6 (31.75-48) AB: 41.3	TD: 6.2 ± 1.68 AB: 6.2 ± 2.1	Male patients aged 18-55 years, with a single distal ureteric stone sized 4-10 mm
Kc et al,^34^ 2016	Nepal	TD: 44 AB: 41	TD 10 mg	Tamsulosin 0.4 mg	2 weeks	TD: 24 AB: 27	TD: 32.0 ± 13.34 AB: 31.4 ± 11.98	TD: 7.1 ± 1.5 AB: 7.1 ± 1.2	Patients aged >18 years, with distal ureteric stones sized 5-10 mm
Kumar et al,^35^ 2015	India	TD: 90 AB: 180	TD 10 mg	Tamsulosin 0.4 mg Silodosin 8 mg	4 weeks	TD: 67 AB: 126	TD: 37.5 ± 13.50 AB: 36.6 ± 10.03	TD: 7.8 ± 1.35 AB: 7.5 ± 1.24	Patients aged >18 years, with a distal ureteric stone sized 5-10 mm, diagnosed by noncontrast CT, or USG KUB
Laddha et al,^36^ 2019	India	TD: 50 AB: 50	TD 10 mg	Tamsulosin 0.4 mg	2 weeks	TD: 39 AB: 34	TD: 45.4 ± 13.16 AB: 43.2 ± 15.52	TD: 6.3 ± 1.4 AB: 6.1 ± 1.4	Patients with a distal ureteric stone sized ≤10 mm
Parikh et al,^2^ 2019	India	TD: 30 AB: 30	TD 10 mg	Tamsulosin 0.4 mg	3 weeks	TD: 21 AB: 22	NA	TD: 7.2 ± 1.5 AB: 7.3 ± 1.28	Patients aged >20 years, with distal ureteric stones sized <10 mm
Puvvada et al,^3^ 2016	India	TD: 100 AB: 100	TD 10 mg	Tamsulosin 0.4 mg	4 weeks	TD: 65 AB: 67	TD: 36.3 ± 11.32 AB: 37.5 ± 12.67	TD: 7.1 ± 1.43 AB: 7.2 ± 1.25	Patients aged ≥18 years, with a distal ureteric stone sized 5-10 mm, diagnosed by noncontrast CT KUB
Rahim et al,^4^ 2020	Egypt	TD: 33 AB: 33	TD 5 mg	Tamsulosin 0.4 mg	4 weeks	NA	TD: 32.1 ± 8.2 AB: 31.91 ± 7.9	TD: 5.5 ± 1.5 AB: 5.9 ± 1.9	Patients aged ≥18 years, with symptomatic distal ureteric stones <10 mm, diagnosed by noncontrast CT KUB

AB, alpha-blocker; CT, computed tomography; KUB, kidney, ureter, and bladder; NA, not available; TD, tadalafil; USG, ultrasound.

^†^Mean or median.

## Data Availability

The data that support the findings of this study are available on request from the corresponding author.
