# Supplementary material for: Tadalafil Versus Tamsulosin or Silodosin as Medical Expulsive Therapy for Distal Ureteral Stones: A Systematic Review and Meta-Analysis of Randomized Controlled Trials
Source: Urol Res Pract. 2025 Dec 5;51(5):179–88. doi: 10.5152/tud.2025.24145 (PMC12771009; doi:10.5152/tud.2025.24145)
Supplement: Supplementary Material [file supplementary_material.pdf]

Study or Subgroup	Tadalafil Events	Tadalafil Total	Alpha-Blockers Events	Alpha-Blockers Total	Weight	Risk Ratio M-H, Random, 95% CI
Abdelaal 2023	7	50	13	100	13.4%	1.08 [0.46, 2.53]
Falahatkar 2021	7	44	2	44	4.2%	3.50 [0.77, 15.92]
Goyal 2018	7	62	6	61	9.2%	1.15 [0.41, 3.22]
Gur 2021	7	46	11	97	12.6%	1.34 [0.56, 3.24]
KC 2016	12	44	6	41	12.5%	1.86 [0.77, 4.51]
Kumar 2015	14	90	18	180	23.0%	1.56 [0.81, 2.98]
Parikh 2019	4	30	3	30	4.9%	1.33 [0.33, 5.45]
Puvvada 2016	14	100	11	100	17.8%	1.27 [0.61, 2.67]
Rahim 2020	9	33	1	33	2.4%	9.00 [1.21, 67.09]
**Total (95% CI)**		**499**		**686**	**100.0%**	**1.50 [1.09, 2.04]**
Total events	81		71			

Heterogeneity: Tau² = 0.00; Chi² = 5.73, df = 8 (P = 0.68); I² = 0%
Test for overall effect: Z = 2.53 (P = 0.01)

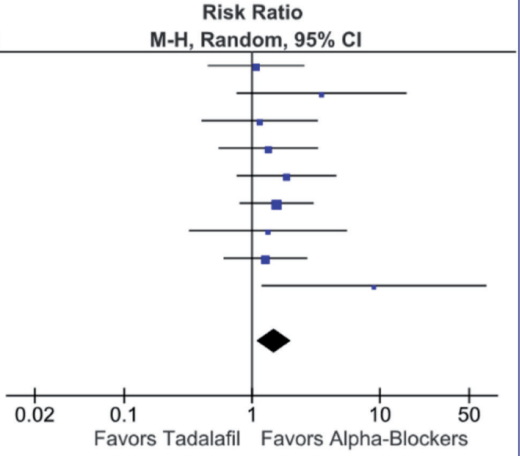

Supplementary Figure 1. The rate of headache was significantly higher in the tadalafil group (p = 0.01).

Study or Subgroup	Tadalafil Events	Tadalafil Total	Alpha-Blockers Events	Alpha-Blockers Total	Weight	Risk Ratio M-H, Random, 95% CI
Abdelaal 2023	3	34	22	67	18.0%	0.27 [0.09, 0.83]
Falahatkar 2021	0	23	3	24	2.7%	0.15 [0.01, 2.73]
Goyal 2018	1	41	6	46	5.4%	0.19 [0.02, 1.49]
Gur 2021	2	46	12	97	10.9%	0.35 [0.08, 1.51]
Kumar 2015	4	67	17	126	21.0%	0.44 [0.16, 1.26]
Parikh 2019	2	21	4	22	9.1%	0.52 [0.11, 2.56]
Puvvada 2016	6	65	12	67	27.3%	0.52 [0.21, 1.29]
Rahim 2020	1	33	7	33	5.6%	0.14 [0.02, 1.10]
**Total (95% CI)**		**330**		**482**	**100.0%**	**0.36 [0.22, 0.59]**
Total events	19		83			

Heterogeneity: Tau² = 0.00; Chi² = 2.79, df = 7 (P = 0.90); I² = 0%
Test for overall effect: Z = 4.13 (P < 0.0001)

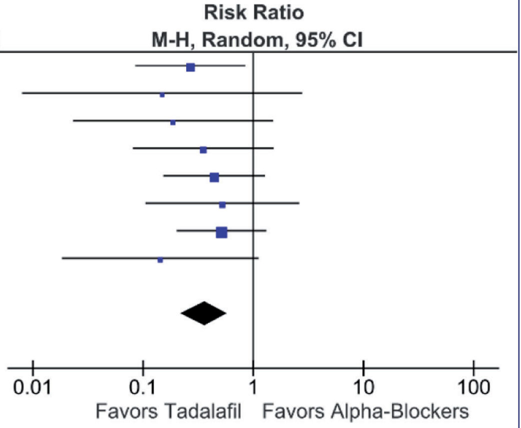

Supplementary Figure 2. The rate of abnormal ejaculation was significantly lower in the tadalafil group (p < 0.001).

Study or Subgroup	Tadalafil Events	Tadalafil Total	Alpha-Blockers Events	Alpha-Blockers Total	Weight	Risk Ratio M-H, Random, 95% CI
Abdelaal 2023	3	50	14	100	9.8%	0.43 [0.13, 1.42]
Falahatkar 2021	5	44	2	44	5.6%	2.50 [0.51, 12.21]
Goyal 2018	6	62	4	61	9.5%	1.48 [0.44, 4.97]
KC 2016	8	44	5	41	13.2%	1.49 [0.53, 4.19]
Kumar 2015	14	90	17	180	32.3%	1.65 [0.85, 3.19]
Parikh 2019	4	30	3	30	7.1%	1.33 [0.33, 5.45]
Puvvada 2016	12	100	10	100	22.5%	1.20 [0.54, 2.65]
**Total (95% CI)**		**420**		**556**	**100.0%**	**1.32 [0.91, 1.93]**
Total events	52		55			

Heterogeneity: Tau² = 0.00; Chi² = 4.61, df = 6 (P = 0.60); I² = 0%
Test for overall effect: Z = 1.47 (P = 0.14)

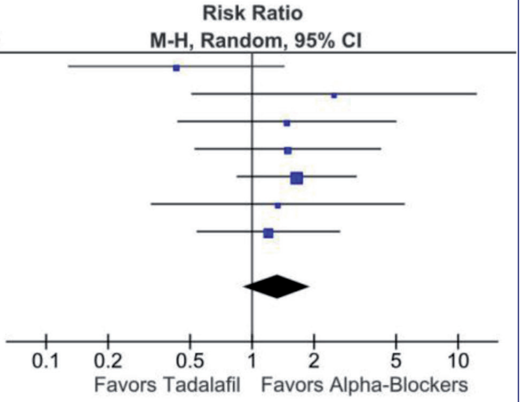

Supplementary Figure 3. There was no significant difference in terms of dizziness.

Study or Subgroup	Tadalafil Events	Tadalafil Total	Alpha-Blockers Events	Alpha-Blockers Total	Weight	Risk Ratio M-H, Random, 95% CI
Abdelaal 2023	4	50	18	100	13.1%	0.44 [0.16, 1.24]
Falahatkar 2021	4	44	1	44	3.7%	4.00 [0.47, 34.38]
Goyal 2018	3	62	3	61	6.7%	0.98 [0.21, 4.69]
Gur 2021	7	46	9	97	15.3%	1.64 [0.65, 4.13]
KC 2016	11	44	4	41	12.5%	2.56 [0.89, 7.41]
Kumar 2015	14	90	17	180	23.3%	1.65 [0.85, 3.19]
Parikh 2019	3	30	4	30	7.9%	0.75 [0.18, 3.07]
Puvvada 2016	9	100	11	100	17.5%	0.82 [0.35, 1.89]
**Total (95% CI)**		**466**		**653**	**100.0%**	**1.22 [0.79, 1.87]**
Total events	55		67			

Heterogeneity: $Tau^2 = 0.09$; $Chi^2 = 9.35$, df = 7 (P = 0.23); $I^2 = 25\%$

Test for overall effect: Z = 0.89 (P = 0.37)

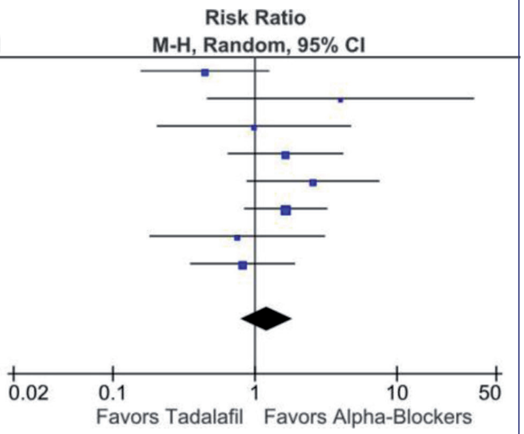

Supplementary Figure 4. There was no significant difference in terms of back pain.

Study or Subgroup	Tadalafil Events	Tadalafil Total	Alpha-Blockers Events	Alpha-Blockers Total	Weight	Risk Ratio M-H, Random, 95% CI
Abdelaal 2023	2	50	19	100	9.2%	0.21 [0.05, 0.87]
Falahatkar 2021	0	44	2	44	2.3%	0.20 [0.01, 4.05]
Goyal 2018	2	62	2	61	5.3%	0.98 [0.14, 6.76]
Gur 2021	3	46	9	97	11.2%	0.70 [0.20, 2.47]
KC 2016	7	44	4	41	12.9%	1.63 [0.52, 5.16]
Kumar 2015	9	90	9	180	19.1%	2.00 [0.82, 4.86]
Parikh 2019	2	30	3	30	6.6%	0.67 [0.12, 3.71]
Puvvada 2016	8	100	10	100	19.1%	0.80 [0.33, 1.94]
Rahim 2020	5	33	6	33	14.2%	0.83 [0.28, 2.46]
**Total (95% CI)**		**499**		**686**	**100.0%**	**0.89 [0.56, 1.41]**
Total events	38		64			

Heterogeneity: $Tau^2 = 0.09$; $Chi^2 = 9.74$, df = 8 (P = 0.28); $I^2 = 18\%$

Test for overall effect: Z = 0.51 (P = 0.61)

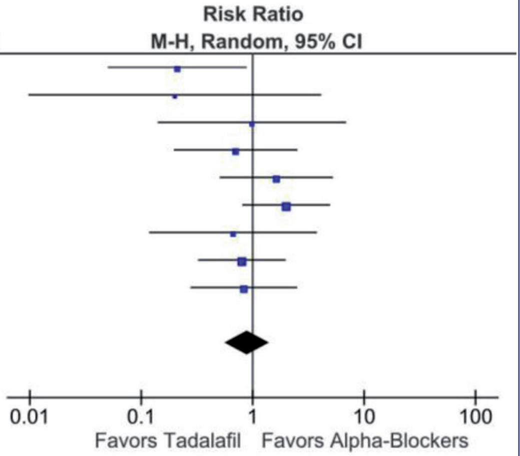

Supplementary Figure 5. There was no significant difference in terms of orthostatic hypotension.

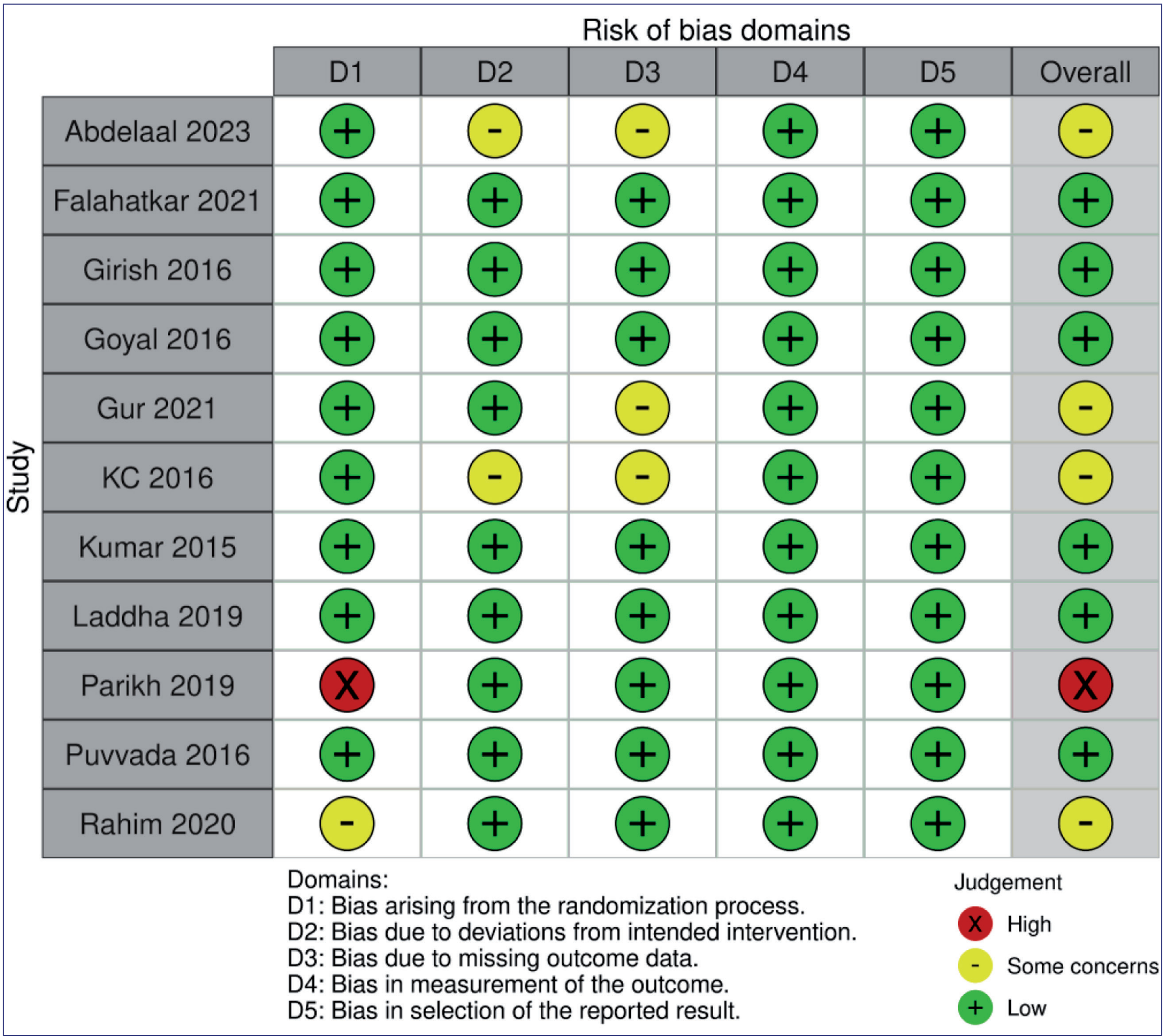

Supplementary Figure 6. Risk of bias assessment.

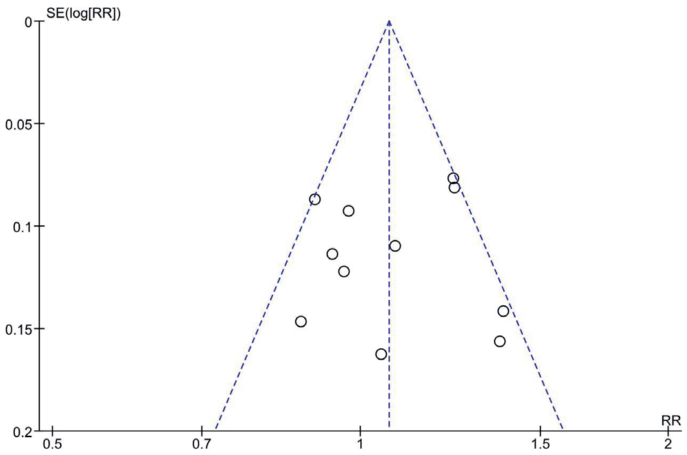

Supplementary Figure 7. Funnel plot for stone expulsion rate.
